# Probabilistic Decision-Making in Children With Dyslexia

**DOI:** 10.3389/fnins.2022.782306

**Published:** 2022-06-13

**Authors:** Christa L. Watson Pereira, Ran Zhou, Mark A. Pitt, Jay I. Myung, P. Justin Rossi, Eduardo Caverzasi, Esther Rah, Isabel E. Allen, Maria Luisa Mandelli, Marita Meyer, Zachary A. Miller, Maria Luisa Gorno Tempini

**Affiliations:** ^1^Department of Neurology, UCSF Dyslexia Center, UCSF Memory and Aging Center, University of California, San Francisco, San Francisco, CA, United States; ^2^Department of Psychology, Ohio State University, Columbus, OH, United States; ^3^Department of Psychiatry, Weill Institute for Neurosciences, University of California, San Francisco, San Francisco, CA, United States; ^4^Department of Neurology, University of California, San Francisco, San Francisco, CA, United States; ^5^Department of Epidemiology and Biostatistics, University of California, San Francisco, San Francisco, CA, United States

**Keywords:** dyslexia, probabilistic, decision-making, risk, reward, striatum, Bayesian

## Abstract

**Background:**

Neurocognitive mechanisms underlying developmental dyslexia (dD) remain poorly characterized apart from phonological and/or visual processing deficits. Assuming such deficits, the process of learning complex tasks like reading requires the learner to make decisions (i.e., word pronunciation) based on uncertain information (e.g., aberrant phonological percepts)—a cognitive process known as probabilistic decision making, which has been linked to the striatum. We investigate (1) the relationship between dD and probabilistic decision-making and (2) the association between the volume of striatal structures and probabilistic decision-making in dD and typical readers.

**Methods:**

Twenty four children diagnosed with dD underwent a comprehensive evaluation and MRI scanning (3T). Children with dD were compared to age-matched typical readers (*n* = 11) on a probabilistic, risk/reward fishing task that utilized a Bayesian cognitive model with game parameters of risk propensity (γ^+^) and behavioral consistency (β), as well as an overall adjusted score (average number of casts, excluding forced-fail trials). Volumes of striatal structures (caudate, putamen, and nucleus accumbens) were analyzed between groups and associated with game parameters.

**Results:**

dD was associated with greater risk propensity and decreased behavioral consistency estimates compared to typical readers. Cognitive model parameters associated with timed pseudoword reading across groups. Risk propensity related to caudate volumes, particularly in the dD group.

**Conclusion:**

Decision-making processes differentiate dD, associate with the caudate, and may impact learning mechanisms. This study suggests the need for further research into domain-general probabilistic decision-making in dD, neurocognitive mechanisms, and targeted interventions in dD.

## Introduction

Developmental dyslexia (dD) is a common neurodevelopmental disorder characterized by difficulty in learning how to read and/or spell. Putative cognitive mechanisms underlying dD include aberrant phonological processing (International Dyslexia Association; Bradley and Bryant, 1983; Lyon et al., 2003), visual processing (Stein and Walsh, 1997; Gori and Facoetti, 2015), and/or integration of visual and phonological information (Provazza et al., 2019). Clinical interventions predicated on these mechanisms have demonstrated moderate success (Al Otaiba and Fuchs, 2006; Snowling and Hulme, 2012; Stevens et al., 2021). This has led researchers to wonder if heterogeneity of pathogenic mechanisms exists within dD (Pennington, 2006; Mascheretti et al., 2018; Stein, 2018; Giofrè et al., 2019; Peters and Ansari, 2019; O’Brien and Yeatman, 2021). An under-explored, yet unifying potential mechanism is inconsistent probabilistic decision-making in the context of uncertain percepts, such as phonological percepts.

Research in dD has primarily focused on learners with reading difficulties that appear to be based in phonological processing (Bradley and Bryant, 1983; Stein, 2018). Less is known about individuals who perform poorly in reading but do not evidence phonological processing difficulties or do not benefit from phonologically oriented interventions. In a recent study by Ring and Black (2018), no cognitive mechanism was identified in more participants with reading deficits (28%) than was a phonological mechanism (23%), although the percentages were similar. Ways to better capture possible heterogeneity and/or novel cognitive factors include subtyping samples based on the presence or absence of phonological difficulties as well as examining novel mechanisms. We assess performance on a probabilistic risk/reward decision-making task, an understudied cognitive process that may relate to how children respond when presented with uncertain information, e.g., phonological information if the child has phonological difficulties or visual information if the child has visual processing difficulties, etc.

Decision-making is integral to the learning process. Most models of learning incorporate decision-making and feedback loops on the outcomes of decisions (see Lee et al., 2012; O’Doherty et al., 2017, for reviews). We theorize that in dyslexia, domain-general processes like decision-making may mediate response selection in the setting of perceptual difficulties. Consider that, generally speaking, differences in decision-making become more apparent as uncertainty in the initial decision state increases, and the amount of uncertainty in an initial decision state changes the learning rate for a decision-maker (Lee et al., 2012). Applying these principles to dyslexia, a student with phonological impairments may experience greater uncertainty when faced with “phonological decisions,” i.e., sounding out syllables in unrecognized words. Moving past such uncertainty requires engaging the complex cognitive process of decision-making, the parameters of which vary across individuals. Such differences in decision-making may influence an individual’s ability to learn from phonological information for a variety of reasons, e.g., limiting the individual’s ability to properly build expected outcomes, accumulate enough information for a decision, determine probabilistic contingencies, rank competing choices, place value on the outcome after a decision, etc. Indeed, two recent studies found inefficient evidence accumulation in dyslexia (Stefanac et al., 2021; Manning et al., 2022); however, to our knowledge, other components of decision-making in dyslexia have yet to be explored.

Decision-making processes independent of perceptual differences have been suggested to play a role in learning disorders, including dD (Lum et al., 2013; Gabay et al., 2015a; Krishnan et al., 2016; Elleman et al., 2019; O’Brien and Yeatman, 2021). Cognitive neuroscience studies suggest that the neuropsychological mechanisms involved in uncertainty play a role in learning (Behrens et al., 2007; Massi et al., 2018). Uncertainty is the state in which a decision-maker has imperfect knowledge of what outcome will follow from a certain choice (Platt and Huettel, 2008). Learning of probabilistic information falls under the umbrella of uncertainty and some studies have found specific difficulties in probabilistic learning in dD (Gabay et al., 2015b; Elleman et al., 2019). Probability is an inherent feature in the English language because of irregular and inconsistent phoneme-grapheme relationships (Hsu and Chater, 2010; Yang et al., 2017). Although the acquisition of reading ability in the context of semi-regular phoneme-grapheme relationships has generally been studied using statistical learning paradigms (Arciuli, 2018), probabilistic learning can unify statistical learning along with other types of learning such as perceptual (Fiser and Lengyel, 2019) as a supraordinate element. Understanding how children with dD approach probabilistic learning for non-linguistic material will provide important insights into the process of learning in the context of uncertainty. Decision-making can also influence the effort exerted to resolve uncertainty and internal motivational states impact valuations during the decision-making process, e.g., outcome valuations based on feedback if the answer was correct or incorrect (O’Doherty et al., 2017). Furthermore, sensitivity to risk and reward has been associated with adaptive learning (McCormick and Telzer, 2017). Given the difficulty children with dD have in reading, understanding how they approach risk/reward situations could be helpful in designing interventions.

Risk/reward tasks are used frequently in decision-making studies and can easily be assessed using probabilistic paradigms and without symbolic representations (a frequent confound in studies of dD) (Lejuez et al., 2002; Pleskac, 2008). When a decision-maker is confronted with a task that has inherent uncertainty they must choose whether or not, or how vigorously to embark on such an endeavor. The decision will be influenced by the anticipated reward and risk (Schultz, 2016). Uncertainty, risk/reward assessments, and motivation-cognition interactions are associated with fronto-striatal brain systems (motivation-cognition interactions; Braver et al., 2014; uncertainty, Daw et al., 2005; Pauli et al., 2016), which have also been posited to develop differently in children with learning disorders but have been less explored than regions associated with language (Krishnan et al., 2016; Hancock et al., 2017; Massarwe et al., 2021). Furthermore, the ventral and dorsal striatum have been found to relate to knowledge acquisition (Pine et al., 2018), risk and reward processing (Preuschoff et al., 2006), and language to a lesser extent (Chan et al., 2013). Activity in the ventral striatum, in particular, has been shown to dynamically change during reward and learning phases in relation to uncertainty and influence motivational continuation of a behavior and selective attention (Heekeren et al., 2007; Bourgeois et al., 2016). Additionally, dopaminergic signals enhance perceptual representation when paired with reward value to increase salience and draw attention to stimuli (Berridge and Robinson, 1998). In particular, dopaminergic signals in the striatum are associated with motivation and reward-based learning (Palmiter, 2008). Therefore, the striatum is a good first candidate for an underexplored neural basis underlying differences that may exist between children with dD and typical children on risk/reward decision-making tasks.

We investigated how children with dD and age-matched controls play a probabilistic risk-reward decision-making game adapted for children with dD from the Angling Risk Task (Pleskac, 2008; Zhou et al., 2021) and explored how cognitive constructs measured by the game (risk propensity and behavioral consistency) are associated with striatal brain structures.

We hypothesized that (1) children with dD would have distinct risk/reward profiles from controls based in their approach to confronting uncertain information, and (2) the risk/reward parameters of the cognitive model of the game would associate with striatal volumes given previous literature linking risk/reward and uncertain decisions to the striatum.

## Materials and Methods

### Participants/Recruitment

Individuals were selected from the recruitment base at University of California, San Francisco (UCSF) Dyslexia Center, a multidisciplinary research program that performs neurological, psychiatric, cognitive and neuroimaging evaluations of children with language-based neurodevelopmental disorders. A diagnosis of dD based on IDA criteria, an age between 7 and 15 years, and fluency in English were necessary for inclusion in the study. Exclusion criteria for the dD group in this analysis included a diagnosis of attention deficit/hyperactivity disorder (ADHD), all objective reading and spelling scores at the time of study falling above the 25th percentile of same aged peers and within 2SD of their estimated general cognitive abilities, general cognitive scores that fell below the 9th percentile of same aged peers (indicative of borderline or lower intellectual functioning), known history of perinatal events such as born prematurely with very low birth weight, an acquired brain injury or tumor, genetic, neurological, or psychiatric disorder associated with seizures, strokes, impaired sensory processing or communication. The 25th percentile (the low end of average) was used as the cut-off for reading scores to increase sensitivity because several children in the dD cohort had received regular or intensive reading intervention or tutoring and therefore may have remediated reading impairment. The intelligence quotient (IQ) discrepancy was also added due to the presence of some children with IQs in the very superior range and the desire to be inclusive in the definition of dD. 24 children with dD met criteria for inclusion in the analysis.

Control participants (*n* = 11) were recruited through local schools and parent networks. Exclusion criteria for controls were any standardized single-word reading scores that fell below the 16th percentile (-1SD) of same aged peers, general cognitive scores that fell below the 9th percentile of same aged peers, and any history of developmental delays, or a known genetic, neurological, or psychiatric disorder that impacts cognition such as ADHD, autism spectrum disorders, or depression. In order to improve control recruitment and retention, control participants underwent an abbreviated study protocol. Table 1 outlines the demographic characteristics of the participants. Guardians of the participants provided informed written consent and participants provided assent. The study was approved by the UCSF Committee on Human Research and complies with the declaration of Helsinki.

**TABLE 1:** Demographic, questionnaire, and testing data.

	Controls	dD	
N	11	24	Sig
**Demographics and behavior**			
Age (cog testing), years	9.94 [1.3]	10.48 [1.5]	0.30
Age (MRI), years	9.50 [1.4]	9.96 [1.5]	0.40
Time between MRI and Cog tests, years	0.48 [0.3]	0.52 [0.3]	0.77
Sex	7 F, 4 M	11 F, 13 M	0.33
Handedness	11 R	21 R, 3 NR	0.22
DSIS (1 = lowest; 5 = highest)			
Parent-reported impulsivity	2.36 [1.0]	2.57 [0.9]	0.58
Child-reported impulsivity	1.79 [0.7]	2.26 [0.7]	0.10
Parent-reported schoolwork	2.19 [1.1]	2.70 [1.2]	0.29
Child-reported schoolwork	1.78 [0.6]	2.60 [0.8]	0.008
Parent-reported interpersonal	2.53 [1.2]	2.44 [1.1]	0.85
Child-reported interpersonal	1.81 [1.0]	1.91 [1.0]	0.78
Vanderbilt inattention sum	–	6.41 [4.3]	N/A
Vanderbilt hyperactivity sum	–	4.17 [3.1]	N/A
		**(% ile)**	
BASC externalizing	–	33.67 [26.2]	N/A
BASC internalizing	–	39.22 [35.0]	N/A
BASC attention	–	37.22 [26.9]	N/A
**Cognition and academics**	**(% ile)**	**(% ile)**	
Matrix reasoning	67.38 [16.8]	59.46 [26.2]	0.43
WJ oral vocabulary	72.25 [19.7]	48.80 [24.0]	0.002
WJ letter-word Identification	–	22.19 [18.6]	N/A
WJ word attack	–	35.25 [25.0]	N/A
TOWRE SWE	47.00 [25.1]	15.52 [20.2]	0.002
TOWRE PDE	53.20 [30.3]	15.13 [18.5]	<0.001
GORT 5 accuracy	53.95 [34.6]	9.35 [11.3]	<0.001
GORT 5 rate	62.45 [26.8]	16.27 [16.7]	<0.001
GORT 5 comprehension	52.30 [21.8]	20.77 [15.9]	<0.001
WJ sound blending	–	54.83 [25.6]	N/A
WJ sound awareness	–	37.50 [25.5]	N/A
WJ rapid picture naming	56.63 [23.5]	33.38 [19.7]	0.01
NEPSY II naming	65.00 [27.7]	36.13 [24.6]	0.01
WJ memory for words	73.13 [24.4]	32.00 [20.7]	<0.001
WISC-IV integr. digit span fwd	64.22 [29.5]	25.63 [22.9]	<0.001
WISC-IV integr. digit span bwd	60.44 [30.8]	26.00 [22.4]	0.001
Rey-O 3’ delay	46.67 [30.1]	38.78 [32.7]	0.60
Children’s colored trails 1	49.14 [24.4]	42.30 [28.1]	0.57
Children’s colored trails 2	45.00 [14.1]	22.80 [20.4]	0.01

### Neuropsychological and Academic Assessment

Neuropsychological and academic testing was administered by research staff or neuropsychology fellows who were trained and supervised by neuropsychologists. Neuropsychological testing covered screening of non-verbal reasoning, processing speed, attention and working memory, verbal and visual recall, visuospatial abilities, and executive functioning (see Table 1 for a full list of tests (Delis et al., 1994; Meyers and Meyers, 1995; Llorente et al., 2003; Wechsler, 2004; Korkman et al., 2007). Academic testing was done using the Woodcock-Johnson IV (WJ-IV) (Schrank et al., 2014). In addition to some of the untimed reading measures in the WJ-IV, participants were also administered the Test of One-Word Reading Efficiency, version 2 (TOWRE-2) and the Gray Oral Reading Test, version 5 (Torgesen et al., 2012; Wiederholt and Bryant, 2012). Impulsivity data were collected via parent and child report on the (Domain-Specific Impulsivity Scale; Tsukayama et al., 2013). Other child behaviors relevant to psychiatric comorbidity were examined through parent responses on the NICHQ Vanderbilt Assessment Scale (NICH Q)^1^ and the Behavior Assessment System for Children-Second Edition (BASC-2; Kamphaus et al., 2007).

### Experimental Task

**FIGURE 1:**
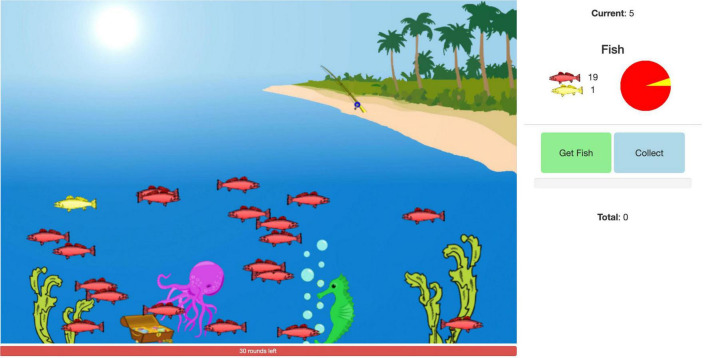
A snapshot of a participant’s view of the task. The participant is asked on every trial to decide to collect the current points (“Collect” button) or go fishing for more points (“Get Fish” button). They can explicitly determine the risk based on three representations: the ratio of yellow to red fish in the pond, the ratio represented in digit form in the legend on the right, and the ratio in the pie chart on the right. Caught red fish earn the player five points. Caught yellow fish result in the subtraction of the current points at the top right.

The angling risk task (ART) (Figure 1) was developed at Ohio State University based on a paper by Pleskac (2008). Instructions for the task were audio-recorded and transcribed. All participants received the same instructions. Control participants could choose audio-recorded, transcribed instructions, or both. Dyslexia participants received the audio-recorded instructions with matching text to ensure comprehension. All participants were queried on their independent ability to comprehend the main components of the task. In brief, participants were told their goal was to earn as many points as possible and that there were two types of fish in a pond. Catching a red fish would earn them five points each; catching a yellow fish would cause them to end their turn and lose the points accumulated during that turn. They would be given 30 turns (rounds/trials) of fishing. During each turn they were given the option to continue fishing or to collect their points. They were warned that they would never know in advance what kind of fish they were going to catch.

For the ART, we pre-generated a sequence of maximum number of casts for all 30 trials, meaning that a yellow fish was going to be caught exactly at the maximum number of casts. The maximum casts were generated by a probabilistic process with a given probability of catching a yellow fish, and the proportion of yellow fish on the screen is set to be equal to this probability. In our task, the probability of catching a yellow fish was set to be 0.05 at the beginning, with an increased increment of 0.05 after every five trials. The corresponding means of maximum casts were the reciprocal of the probabilities. Three equivalent versions (A, B, C) of the task were created in which the order of maximum number of casts was reorganized within probability levels so that participants would have the same maximum number of casts but in a different sequence. We conducted a Bayesian ANOVA (Rouder and Morey, 2012) to check the effect of version and the results indicated that the versions are very unlikely to have affected the outcome measures (BF_10_ = 0.309, 0.396, and 0.201 for the adjusted score, γ^+^, and β, respectively). The adjusted score is the average number of casts, excluding trials on which a yellow fish was caught. The other outcome measures, γ^+^, and β are explained below in computational modeling.

### Computational Modeling of Decision Making Processes

The 2-parameter model proposed by Pleskac (2008) and van Ravenzwaaij et al. (2011) was designed to model the cognitive process underlying the Balloon Analog Risk Task (BART; Lejuez et al., 2002), on which the ART is based. The two parameters in this model, γ^+^ and β, represent risk-taking propensity and behavioral consistency, respectively. According to this model, before the start of each trial *k*, the participant has in mind a number of casts (or the points equivalent to a number of casts, e.g., 50 points = 10 casts) deemed as optimal in terms of maximizing the final score in the task, which, denoted as ω_*k*_, is calculated based on this participant’s propensity for risk-taking, γ^+^, and the probability that a yellow fish, which causes the loss of accumulated points on a respective trial, will be caught, $p_{k}^{y\,e\,l\,l\,o\,w}$:


(1) $$\omega_{k}=\frac{-\gamma^{+}}{\ln\left(1-p_{k}^{y\,e\,l\,l\,o\,w}\right)}\;\left(\gamma^{+}\geq 0\right)$$

In the ART task, $p_{k}^{y\,e\,l\,l\,o\,w}$ was represented by the proportion of yellow fish on the screen, which is equal to the underlying probability of catching a yellow fish. Given the $p_{k}^{y\,e\,l\,l\,o\,w}$, participants with a higher risk-taking propensity γ^+^ will have a higher ω_*k*_ in mind compared to less risk-taking participants. On the other hand, given a fixed γ^+^, as the probability of catching a yellow fish increases, the optimal number of casts decreases, i.e., it becomes less likely to obtain a high number of casts before incurring a loss.

**FIGURE 2:**
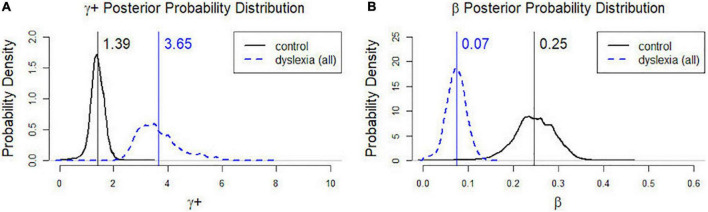
Posterior probability distributions of γ^+^ **(A)** and β **(B)** in the control group and the dD group. γ^+^: Control mean = 1.39, CI: 0.78, 1.90; Dyslexia mean = 3.65, CI: 2.49, 5.64. β:Control mean = 0.25, CI: 0.14, 0.33; Dyslexia mean = 0.07, CI: 0.03, 0.12.

The model also takes account of the fact that human behavior can be random to some extent by incorporating a logistic response function that calculates the probability that a participant is going to cast given opportunity *l* (= 1,2,…) during trial *k*, denoted as $p_{k\,l}^{c\,a\,s\,t}$:


(2) $$p_{k\,l}^{c\,a\,s\,t}=\frac{1}{1+e^{\beta \,\left(l-\omega_{k}\right)}}\;\left(\beta \geq 0\right)$$

Therefore, with each opportunity to cast, the participant’s decision (to cast or collect) follows a Bernoulli distribution (a binomial probability distribution when choices are binary) with the probability of casting equal to $p_{k\,l}^{c\,a\,s\,t}$. With a given ω_*k*_ in mind, the probability of casting decreases as *l* increases, indicating that with each cast, the probability of making another cast decreases. When *l* is equal to ω_*k*_, the probability of casting is 0.5 (i.e., chance level), indicating that before *l* reaches ω_*k*_, the probability of casting is larger than chance, and when *l* has passed ω_*k*_, the probability of casting is smaller than chance. Behavioral consistency, β, characterizes the extent to which the participant makes casting decision based on ω_*k*_. A lower β value indicates that the participant’s casting decision is more random. In an extreme scenario in which β is equal to zero, the probability of casting is equal to 0.5, regardless of the opportunity *l* value and the optimal number of casts ω_*k*_,. As β gets larger, the decision of whether to cast or not becomes more deterministic. For example, for β = 0.5, if a participant wanted to cast 10 times to obtain 50 points for the first trial (*k* = 1), ω_1_ = 10, then for the first opportunity (*l* = 1), the probability that the participant will cast. $p_{k=1,l=1}^{c\,a\,s\,t}$ = $\frac{1}{1+e^{\left(0.5\right)\,\left(1-10\right)}}$ would be equal to 0.989 or close to a 100% probability that the participant will cast. When opportunity increases (i.e., the participant makes more casts), this probability decreases, until a yellow fish is caught or a decision to collect is made.

**FIGURE 3:**
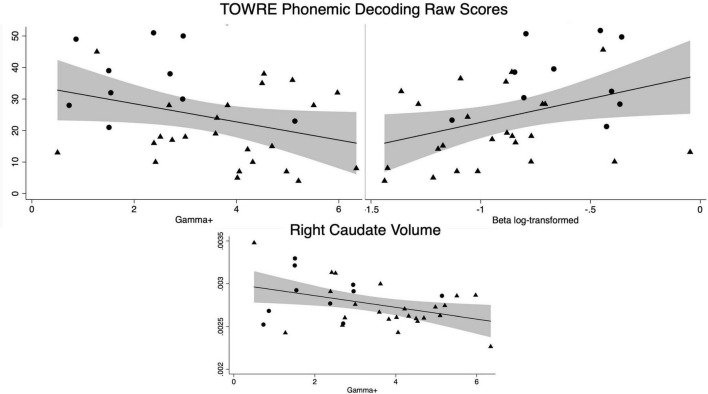
Significant correlations across groups of model parameters with reading and the right caudate volume (adjusted for total intracranial volume). Circles represent controls and triangles represent dD participants. The shaded area around the linear fit represents the 95% confidence interval.

### Striatal Volume Segmentation

All but two subjects underwent a brain MRI acquisition on a 3T Siemens Prisma scanner (Siemens, Erlangen, Germany) equipped with a 64-channel head/neck coil. A high resolution T1-weighted three-dimensional was acquired for each participant within 6 months of cognitive evaluation. The Magnetization Prepared Rapid Acquisition Gradient Echo (MPRAGE) sequence consists of the following parameters: 160 sagittal slices, an isotropic voxel of 1 mm size, TR/TE/TI = 2,300/2.9/900 ms, Field of view (FOV): 256 × 256, flip angle = 9 deg, parallel imaging acceleration factor (iPAT) = 2. Images were visually inspected for quality check purposes and to exclude the presence of artifacts and brain abnormalities, which includes visual review for ghosting, ringing, aliasing, and excessive head movement. In the presence of visible motion artifacts at data collection, the sequence is repeated to obtain better quality. Mild motion not visually detected at data collection is corrected through the Freesurfer (version 5.3)^2^ recon-all package.^3^ All of the images included in this study passed the quality checks.

Automatic segmentation of the caudate, putamen and nucleus accumbens bilaterally was obtained with FreeSurfer. The T1-weighted images were processed through the standard processing, which involved non-uniform intensity normalization, removal of non-brain tissue, affine registration to the Montreal Neurological Institute (MNI) space and Talairach transformation, and segmentation of gray/white matter tissue. Segmented images are visually inspected when there are anomalous values reported for the volume metrics, e.g., the scale is different from all other subjects. None of the segmented images in this analysis required manual inspection. Subcortical parcellation was performed by using the Desikan-Kyliany Atlas (Desikan et al., 2006) and, the results were visually inspected by a trained neuroradiologist. Volumes of interest (VOI) were calculated through voxel count within the region of interest and then multiplied by voxel size to obtain the volume. The VOI was corrected by the total intracranial volume (TIV) for the following statistical analysis.

### Statistics

Demographic, cognitive and gray matter region of interest data were analyzed using Stata 15 (StatCorp, College Station, TX). Independent group Student’s *t*-tests and chi-squared analyses were used for parametric and non-parametric data, respectively, using tests for unequal variances as appropriate. All pairwise and multiple comparisons used a Bonferroni correction. Pearson’s correlations were used for associations between parameter estimates and raw reading scores and striatal TIV-corrected volumes. Permutation tests were completed to examine sample effects when appropriate (Proschan et al., 2014).

We used Bayesian estimation to obtain the posterior probability distributions of the parameters and used the means as point estimates in statistical analyses. The Bayes factor (BF) characterizes the strength of the evidence for the alternative hypothesis over the null hypothesis. Values smaller than one suggest that the observed data favor the null hypothesis, and values larger than one favor the alternative hypothesis. An approximate classification scheme of how to interpret BFs can be found in Supplementary Material. Values between 1 and 3 are considered anecdotal evidence in favor of the alternative. Values between 3 and 10 are moderate evidence, and greater than 10 provide strong evidence.

Bayesian analyses were conducted using JASP statistical software [JASP Team, 2020, JASP (Version 0.14.1) (Computer software)]. Bayesian independent samples *t*-tests were conducted to compare the adjusted game scores in the Control group and the dD group. In order to estimate and compare the two model parameters (γ^+^, β) in Eqs (1) and (2) on a group level, Bayesian analysis was extended to create a hierarchical Bayesian model (Gelman et al., 2014). This method allows for the estimation of individual-level parameter distributions as well as group-level model parameter distributions (Lee, 2011). The hierarchical Bayesian model was implemented using the RStan R package (Stan Development Team, 2020). The model code can be found in Supplementary Material.

## Results

### Demographics

There were no group (dD vs. controls) differences based on age, sex, or handedness. The average age (±*SD*) of participants was 10.31 ± 1.40 years with a range from 7.99 to 12.63 years. The age range was 8.30–11.61 years for controls and 7.99–12.63 years for dD. There were 18 females and 17 males included in the analysis. All but three participants were right-handed. More demographics are reported in Table 1.

### Parent and Self-Report Measures

No group differences were found in overall impulsivity in either parent-report or self-report (Domain-Specific Impulsivity Scale) (Table 1). There were also no significant differences between the parent and child report on overall impulsivity. Average parent report of overall impulsivity was 2.51 ± 0.94 (max = 5) and average child report of overall impulsivity was 2.13 ± 0.72. Parent-report of schoolwork-related impulsivity was similar across groups (Average = 2.54 ± 1.18). There were no parent-child differences in reported levels of schoolwork impulsivity, i.e., parents and children had similar perceptions of their schoolwork impulsivity. There were no group differences in parent (Average = 2.47 ± 1.11) or child-reported (Average = 1.88 ± 0.97) interpersonal impulsivity. Parent and child-report of interpersonal impulsivity were similar.

Parents of control children were not asked to complete the Vanderbilt or BASC-2 questionnaires given that these are clinical questionnaires, so we do not have data for between group comparisons. Also, no children in this sample met the symptom thresholds for ADHD inattentive, hyperactive, or combined type on the Vanderbilt.

### Cognitive Testing

Groups did not differ on a non-verbal reasoning measure (Matrix Reasoning), which suggests a similar level of general cognitive abilities. The mean Matrix Reasoning percentile score was 61.44 ± 24.17, which falls in the average range. Similarly, there were also no group differences in visuoconstructional abilities (Rey-O Copy), visual memory (Rey-O delay), or processing speed (Children’s Colored Trails 1); group averages for these measures broadly fell in the average range. As expected, there were significant group differences between children with and without dD on reading measures (TOWRE and GORT), and measures known to associate with dD such as expressive vocabulary and verbal short-term and working memory (WJ Oral Vocabulary and Memory for Words and WISC Digit Span). Other cognitive testing data can be found in Table 1.

### Main Outcome Measures

The low Bayes Factor (BF = 0.347) suggests the two groups do not differ on the overall performance measure of the decision-making task, the adjusted score [Control: mean = 5.28, *SD* = 2.11, (2.5%, 97.5%) quantiles = (2.17, 10.17); dD-phono: mean = 5.05, *SD* = 2.24, (2.5, 97.5%) quantiles = (3.14, 10.10); dD-other: mean = 5.31, *SD* = 2.42, (2.5%, 97.5%) quantiles = (1.91, 9.14)]. A Bayesian ANOVA on the control group and two sub-groups of dD also indicated that there was no group effect in the adjusted scores (BF_10_ = 0.355).

#### Cognitive Model Parameters

As described earlier, the model parameters (γ^+^, β) in Eqs (1) and (2) on a group level were estimated by fitting a hierarchical Bayesian model. The estimated parameter values all converged to their target posterior distributions (see Supplementary Figure 1). The 95% credible intervals (CIs) for γ^+^ were (0.78, 1.90) for the control group and (2.49, 5.64) for the dD group; the 95% credible intervals for β were (0.14, 0.33) for the control group and (0.03, 0.12) for the dD group. The CIs of both model parameters did not overlap between groups, suggesting that the distributions for the two groups were distinctly different in both γ^+^ and β. Figure 2 displays the posterior probability distributions of γ^+^ and β in the control and dD groups, with the posterior means indicated by vertical lines. As suggested by the CIs, the dD group had higher risk-taking propensity (γ^+^) and lower behavioral consistency (β) than the control group.

### Associations Between Cognitive Parameters and Cognitive Variables

Given that the groups differed on the cognitive parameters and cognitive variables related to dD (verbal short-term and working memory), we investigated the relationship between the cognitive parameters and digit span forward and backward. First, we investigated across participants to determine if there was a relationship across diagnostic groups. There were no significant relationships across groups (γ^+^ (risk-taking) and β log transformed, *n.b.* β was log-transformed due to positive skew, *n* = 33, all *p*’s > 0.16) or within the control group (*n* = 10, all *p*’s > 0.5). Within the dD group, there was a trend for a significant relationship between verbal short-term memory (Digit Span Fwd) and both cognitive parameters [*n* = 23, γ^+^ (risk-taking): *r* = 0.37, *p* = 0.08 and β log transformed (consistency): *r* = −0.36, *p* = 0.09]. No relationship was found with verbal working memory (Digit Span Backward) and the cognitive parameters within the dD group (all *p*’s > 0.2).

### Associations With Reading

Both controls and dD participants received timed single-word reading measures. There was a significant relationship in timed single-word pseudoword reading with both the risk-taking parameter and the behavioral consistency parameter when examined across all participants (TOWRE Pseudoword Decoding Efficiency Raw Score (*n* = 34), γ^+^: *r* = −0.33, *p* = 0.05; β log transformed: *r* = 0.38, *p* = 0.03, *n.b.*β was log-transformed due to positive skew). No significant relationships were found in timed single-word sight word reading across all participants (*p*’s > 0.2) or when relationships were studied within groups for either timed single word reading measures (control *p*’s > 0.5; dD *p*’s > 0.3).

Only dD participants received the untimed single word reading measures, WJ Letter-Word Identification and Word Attack; there was no overall significant relationship between either model parameter and the WJ reading measures [γ^+^ (risk-taking) and Letter-Word: *r* = −0.18, *p* = 0.39; γ^+^ and Word Attack: *r* = −0.09, *p* = 0.68]; β (behavioral consistency, *n.b.* β was log-transformed due to positive skew) and Letter-Word: *r* = 0.20, *p* = 0.34; β log transformed and Word Attack: *r* = 0.15, *p* = 0.49).

### Associations With Striatal Volumes

Overall, there were no group or subgroup differences in striatal volumes. Across all participants, there was a significant relationship between the risk-taking model parameter, γ^+^, and the right caudate (*n* = 34: *r* = −0.40, *p* = 0.02). There was also trending relationships between γ^+^ and the left caudate (*n* = 34: *r* = −0.31, *p* = 0.08), γ^+^ and the right nucleus accumbens (*n* = 34: *r* = −0.33, *p* = 0.06), and β (behavioral consistency) and the right caudate (*n* = 34: *r* = 0.32, *p* = 0.07). There were no other significant associations between striatal volumes and the model parameters across all participants (*p*’s > 0.1) or in only control participants (all *p*’s > 0.4).

However, striatal associations were found within the dD group. γ^+^ (risk-taking) was negatively associated with the caudate (left: *r* = −0.42, *p* = 0.04; right: *r* = −0.46, *p* = 0.03) and the accumbens trended toward a negative association (left: *r* = −0.34, *p* = 0.10; right: *r* = −0.37, *p* = 0.07). The putamen was not associated with γ^+^(all *p*’s > 0.3). There were no significant striatal relationships found with β (behavioral consistency) in the dD group (all *p*’s > 0.1).

## Discussion

Children with developmental dyslexia (dD), in the absence of ADHD, demonstrated similar overall task performance but increased risk-taking tendencies and less behavioral consistency than typically developing children on a risk/reward decision-making task that did not involve linguistic materials. Striatal volumes per se did not differentiate groups. However, there were clear differences in associations between striatal volumes and game model parameters between dD and controls. These results suggest that differences in probabilistic decision-making separate children with dD from those without. Decision-making profiles and brain-behavior relationships could potentially be cognitive and biological markers for different instructional or psychopharmacological interventions in dD.

The cognitive model parameter estimates (risk propensity and behavior consistency) differed between control and dyslexic children in interpretable ways, while the overall performance measure, i.e., the adjusted score, yielded no differences. A possible reason for the insensitivity of the adjusted score in our case could be that the maximum numbers of casts in the ART were adjusted to be lower (*M* = 8.16) than the traditional design of this task (*M* = 64) to avoid fatigue (Pleskac, 2008). With this smaller range, the distributions of the adjusted scores could have been compacted, obscuring group differences. Despite this restriction, groups differed in other cognitive model parameters, which the model was sensitive to, including the mean of the maximum number of casts, which is equal to the reciprocal of $p_{k}^{y\,e\,l\,l\,o\,w}$. In our task, the $p_{k}^{y\,e\,l\,l\,o\,w}$ is larger than the tasks using a traditional design, and the estimation of γ^+^ is adjusted accordingly. Therefore, a potential advantage of using computational modeling is that the parameter estimates can be comparable even with different task designs, and are less influenced by the task designs, given that the design variable is taken into account by the model, compared to the behavioral measures.

Another advantage of using the modeling approach is that it is more sensitive to information about the cognitive processes during the ART than the overall performance measure. The two parameters, risk-taking propensity (γ^+^) and behavioral consistency (β) characterize both the underlying psychological variable of interest (risk), and the fact that human behavior can be random to some extent (behavioral consistency). Therefore, it is also possible that the groups did not differ on the adjusted score because of their differences in style of play. For example, one player could consistently collect points around the average for the game (5 casts). A different player could behave inconsistently such that his/her performance could vary greatly from as many as 44 casts on one trial to as little as 0 casts on another trial but average out to five casts as well.

The group differences we found in these two parameters better inform our understanding of probabilistic risk/reward decision-making in children with and without dD. This finding suggests that cognitive modeling can provide a deeper understanding of alternative cognitive processes than tasks designed to only examine impairments. This type of sensitivity can be particularly useful in learning disorders when impairments tend to be focal and global cognition is normal. It also provides more specific information on individuals and how they approach a task such that interventions can be more precise, e.g., if a child has perceptual difficulties and has a high degree of behavioral inconsistency in a probabilistic task like this then it is possible that this student may appear to not benefit from interventions simply because he/she is applying random strategies to deal with the uncertainty of the perceptual information. Indeed, a frequent observation from educational interventionists is that dD students seem to learn how to correctly read a certain word on one page and then read it differently on the next page. It is possible then that future interventions for dD could incorporate teaching children how to identify when they feel uncertain and then routinely, systematically approach the uncertain information until this process becomes habitual.

Studies on decision-making in dD have inconsistent results, most likely due to methodological differences (Banai and Ahissar, 2018; Elleman et al., 2019; Gabay et al., 2015a,b) and possibly because they aim to find group differences in task performance rather than different cognitive approaches, or some combination of these factors. The two decision-making processes most studied in relation to dD are statistical learning and category learning (Sawi and Rueckl, 2019; van Witteloostuijn et al., 2019) and most use linguistic or symbolic information which confounds the interpretation because difficulties in linguistic and symbolic translation are found in dD (Ziegler et al., 2010; Snowling and Melby-Lervåg, 2016). Our study does not fall under the umbrella of statistical learning because our probabilities changed every five trials and we utilized Bayesian statistics in an adaptive design to reach convergence within 30 trials. Additionally, our study is not a category learning study because we did not offer cues, only explicit probabilities, that could help a decision-maker determine when a negative outcome was likely to be imposed. On every trial in our task, the decision-maker has the possibility to collect points within a certain range, so there is no correct or incorrect categorization. We selected a probabilistic decision-making paradigm because it could be done quickly and based on our experience, it most closely represents how children make decisions when they are uncertain. Therefore, we would not expect our results to be interpreted in the same vein as statistical or categorical learning studies. However, similar to Gabay et al. (2015b) and Massarwe et al. (2021), we found that those with dD differ in their responses to probabilistic outcomes. Therefore, our study adds to the literature by demonstrating a spectrum of performance and shows that there can be a general difference, but not impairment, in probabilistic decision-making under certain conditions, e.g., non-linguistic, that may extend to impairments under other conditions, e.g., linguistic.

The relative contribution of probabilistic assessment vs. effects of risk propensity and behavioral consistency per se is difficult to assess, e.g., it is possible that ambiguous terms may modify the distributions of risk-propensity and behavioral consistency. In addition to the study on probabilistic learning in dD (Gabay et al., 2015b), limited evidence supports a link between adolescent risk-taking behaviors and learning disorders (McNamara et al., 2008; Poon and Ho, 2016). However, risk-propensity in childhood has not been widely studied (Bell et al., 2019) and to date no studies have examined risk-propensity in children with dD in the absence of ADHD. We propose that children with dD differ from typical readers in approach on the present task because they make different probabilistic assessments. However, it is also possible that they have greater risk-propensity or lower behavioral consistency, or a combination of these factors even when probabilistic terms are modified. It is currently unclear whether or not this behavioral profile is present before the introduction of reading (i.e., could contribute to the development of a reading disorder) or if it is a learned behavior in response to addressing the uncertainty associated with reading. Our inclination is that both perceptual and decision-making differences are present early on and that it is a confluence of these factors that contribute to persistent reading impairments. It will be important in future work to elucidate the relative contribution of these factors, both on group and individual levels. Such knowledge could be of clinical utility in developing instructional interventions for reading strategies.

Due to our small size, we only examined a few cognitive relationships with the cognitive parameters. We did not find any significant relationships across groups or within groups. However, there was a trend for a relationship between the parameters and verbal short-term memory. The relationship was in the opposite direction of what we anticipated, which suggests that it could be a spurious relationship, a heterogeneous relationship that in our sample trended toward insignificance, or that we do not yet have a full conceptualization of how the cognitive model parameters might relate to standardized cognitive variables. The trend was for a positive relationship between risk-taking and verbal short-term memory and a negative relationship with consistency and verbal short-term memory in the dD group, which is to say that the dD children with higher short-term memory were more likely to take risks in the game and be less consistent in their play. One speculation is that being able to hold information in their short-term memory allowed the children to feel more comfortable taking risks at certain times. However, we are hesitant to speculate further given the weak signal and sample size. Larger studies that examine the relationships between these cognitive parameters and standardized cognitive measures would allow for more substantive interpretation.

Timed pseudoword reading scores were positively correlated with the consistency parameter and negatively correlated with the risk parameter across groups. Timed sight word reading was not associated with either cognitive parameter. One of the main differences between these two lists of words is that one can be decoded using consistent rules (pseudoword reading) and the other cannot. It makes sense that if a child behaves more inconsistently that they would have trouble consistently applying a rule to reading. One of the most common frustrations we hear from educators is that children with reading difficulties will appear to learn a word, read it correctly, and then read it incorrectly in the next paragraph. Another observation is that the children with reading difficulties will not consistently read a word incorrectly. They will make different types of errors on the same word. Given that we only found this association across groups it is possible that we were underpowered to find significant associations within groups, that there is heterogeneity within groups, or that this association relates to reading ability and is not necessarily phenotypic of only children with dyslexia, i.e., it is found on a continuum that spans ability levels.

We did not find any group differences in striatal volumes corrected for total intracranial volume. Striatal differences in dD have been inconsistently reported and tend to be small, so these results are consistent with previous studies (Linkersdörfer et al., 2012; Richlan et al., 2013; Hancock et al., 2017). However, we found inverse striatal-cognitive relationships across groups between the right caudate and the risk-taking parameter. There were also trending relationships with the left caudate, the right nucleus accumbens, and the risk taking parameter. These were likely driven by the associations found within the dD group only. We did not find any results with the putamen. The dissociation between striatal regions was unexpected and is intriguing because there are several studies that describe functional differences between these striatal regions (Grahn et al., 2009; Brovelli et al., 2011). In brief, the nucleus accumbens is involved in reward processing; the caudate is involved in the cognitive planning for goal-directed behaviors; the putamen is involved in forming habits through instrumental learning. Additionally, the putamen is a region that has been associated with language processing and is differentially functionally connected in dD (Booth et al., 2007; Viñas-Guasch and Wu, 2017; Wang et al., 2019). The inverse associations with the caudate and nucleus accumbens may point toward a neurocognitive mechanism related to motivational factors that influence a student’s academic behavior and/or a cognitive approach to tasks that are challenging due to uncertainty related to probabilities, e.g., selecting between competing choices on exams. If this finding is replicated and further elucidated, it could help to inform different approaches to reading interventions, assessments, and individual differences within dD.

Additionally, our sample is small and unequal in group sizes, therefore it would be helpful to replicate these findings in larger studies and with reliability metrics for the cognitive task (Frey et al., 2017; Hedge et al., 2018; Palminteri and Chevallier, 2018). In the future, it would be informative to acquire functional imaging during game play to determine if these cognitive differences arise at different points throughout game play and if there are distinct functional networks active during game play.

In summary, dD is associated with differences in probabilistic decision making compared to typical readers. We demonstrate a compelling dissociation between typical readers and those with dD in cognitive model parameters, which provides an initial step in examining the role that probabilistic decision-making and learning play in dD. Finally, cognitive modeling paradigms may prove to be powerful tools in precision assessment, identification of brain-behavior relationships, and development of targeted interventions for focal developmental disorders like dD.

## Author’s Note

Developmental dyslexia (dD) is a neurodevelopmental disorder that remains a conundrum for researchers and practitioners notwithstanding a canonical mechanism related to phonological processing. Questions of other mechanisms, including domain-general vs. specific mechanisms, persist. The present study addresses these quandaries by using the robust design of a Bayesian cognitive modeling paradigm of probabilistic decision-making to better understand if anomalous decision-making (domain-general) exists in dD. The dD group performed the task at the same level as controls but was more likely to take risks and behave inconsistently. Furthermore, the cognitive model parameters associated with reading and the caudate. This suggests domain-general neurocognitive differences could influence learning in dD. This study exemplifies a precise investigation of distinct, alternative neurocognitive processes that likely contribute to dD.

## Data Availability Statement

The raw data supporting the conclusions of this article will be made available by the authors, without undue reservation.

## Ethics Statement

The studies involving human participants were reviewed and approved by University of California, San Francisco Institutional Review Board. Written informed consent to participate in this study was provided by the participants’ legal guardian/next of kin.

## Author Contributions

CP, MP, JM, and MG contributed to conceptualization of the study. CP, RZ, MP, JM, and MG designed the study. CP, RZ, MP, JM, ER, and MM were involved in the implementation of the study. CP, RZ, MP, JM, EC, and IA analyzed the data. CP, RZ, MP, JM, PR, EC, and IA wrote parts of the manuscript. MLM, ZM, and MG edited the first draft of the manuscript. All authors contributed to manuscript revision, read, and approved the submitted version.

## Conflict of Interest

The authors declare that the research was conducted in the absence of any commercial or financial relationships that could be construed as a potential conflict of interest.

## Publisher’s Note

All claims expressed in this article are solely those of the authors and do not necessarily represent those of their affiliated organizations, or those of the publisher, the editors and the reviewers. Any product that may be evaluated in this article, or claim that may be made by its manufacturer, is not guaranteed or endorsed by the publisher.

## Footnotes

1
https://www.nichq.org/

2
http://surfer.nmr.mgh.harvard.edu/

3
https://surfer.nmr.mgh.harvard.edu/fswiki/recon-all
