# Supplementary material for: Probabilistic Decision-Making in Children With Dyslexia
Source: Front Neurosci. 2022 Jun 13;16:782306. doi: 10.3389/fnins.2022.782306 (PMC9235406; doi:10.3389/fnins.2022.782306)
Supplement: Supplementary file 1 [file Data_Sheet_1.docx]

Supplementary Material

# Supplementary Figures and Tables

**Supplemental Table 1.** A classification scheme for the interpretation of BF_10_ (Lee & Wagenmakers, 2013; Jeffreys, 1961). *Note.* $\mathcal{H}_{0}$ is the null hypothesis and $\mathcal{H}_{1}$ is the alternative hypothesis

| Bayes factor (BF_10_) | Interpretation |
| --- | --- |
| > 100 | Extreme evidence for $\mathcal{H}_{1}$ |
| 30 – 100 | Very strong evidence for $\mathcal{H}_{1}$ |
| 10 – 30 | Strong evidence for $\mathcal{H}_{1}$ |
| 3 – 10 | Moderate evidence for $\mathcal{H}_{1}$ |
| 1 – 3 | Anecdotal evidence for $\mathcal{H}_{1}$ |
| 1 | No evidence |
| 1/3 – 1 | Anecdotal evidence for $\mathcal{H}_{0}$ |
| 1/10 – 1/3 | Moderate evidence for $\mathcal{H}_{0}$ |
| 1/30 – 1/10 | Strong evidence for $\mathcal{H}_{0}$ |
| 1/100 – 1/30 | Very strong evidence for $\mathcal{H}_{0}$ |
| < 1/100 | Extreme evidence for $\mathcal{H}_{0}$ |

**Supplemental Table 2**. Descriptive Statistics of the Posterior Distributions of $\gamma^{+}$ and $\beta$. *Note.* SD = standard deviation. 95% LB = 95% credible interval lower bound. 95% UB = 95% credible interval upper bound.

| Group |  | Mean | SD | 95% LB | 95% UB |
| --- | --- | --- | --- | --- | --- |
| Control | $\gamma^{+}$ | 1.392 | 0.278 | 0.778 | 1.901 |
|  | $\beta$ | 0.246 | 0.048 | 0.144 | 0.330 |
| Dyslexia-all | $\gamma^{+}$ | 3.652 | 0.813 | 2.490 | 5.637 |
|  | $\beta$ | 0.074 | 0.022 | 0.026 | 0.117 |
| dD-phono | $\gamma^{+}$ | 3.005 | 0.955 | 1.119 | 5.235 |
|  | $\beta$ | 0.095 | 0.038 | 0.013 | 0.166 |
| dD-other | $\gamma^{+}$ | 5.690 | 1.986 | 2.416 | 9.570 |
|  | $\beta$ | 0.040 | 0.019 | 0.006 | 0.084 |

**Supplementary Figure 1*.*** Box plots of the main outcome measures: adjusted score and model parameters for risk propensity (Gamma+ or $\gamma^{+}$) and behavioral consistency (Beta or $\beta$).

## Supplementary Figure 2*.* Trace plots of group-level parameters for the control, dD-phono, and dD-other groups. The trace plots indicate that the MCMC samples are well mixed and converged, although the dD-other group performed somewhat worse than the other two groups, which is in line with the high standard deviation seen in the posterior distributions in this group.

**Supplementary Code 1.** The code for the hierarchical Bayesian model (BART_2par_HBA.stan).
