# Supplementary figures and images for: Probabilistic Decision-Making in Children With Dyslexia
Source: Front Neurosci. 2022 Jun 13;16:782306. doi: 10.3389/fnins.2022.782306 (PMC9235406; doi:10.3389/fnins.2022.782306)

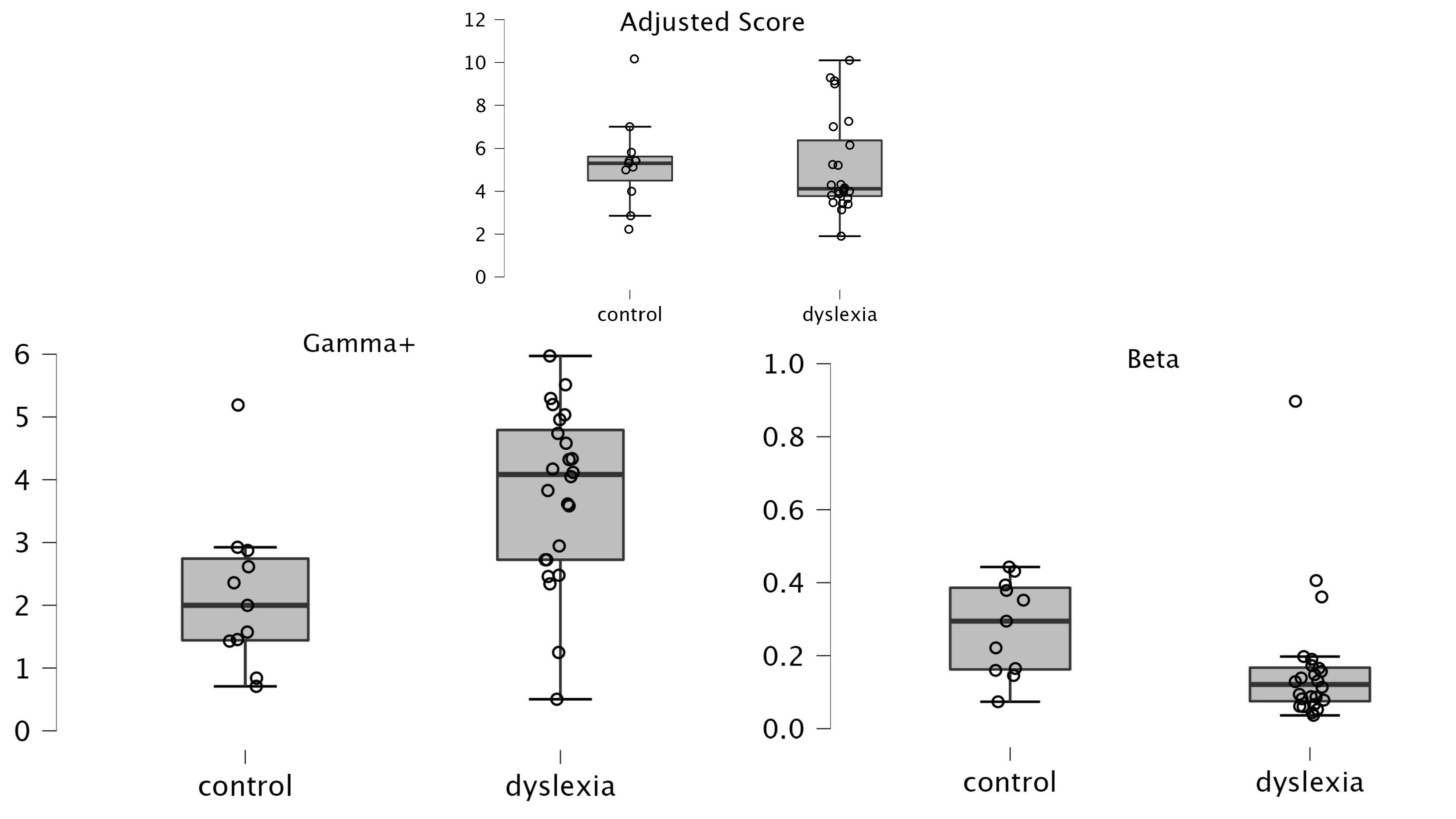

Supplement: Supplementary file 2 [file Image_1.JPEG]

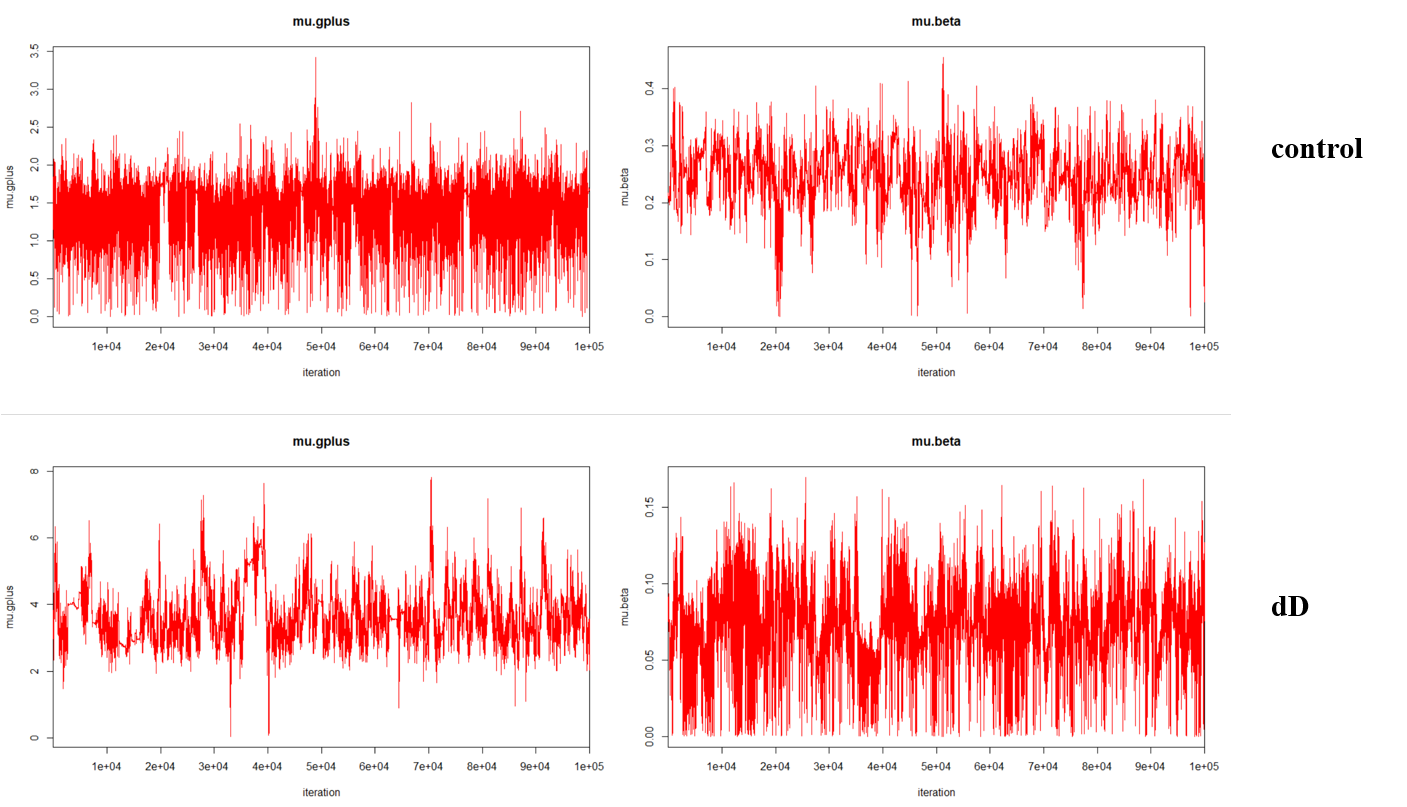

Supplement: Supplementary file 3 [file Image_2.png]
